# Electrostatic Modifications of the Human Leukocyte Antigen-DR P9 Peptide-Binding Pocket and Susceptibility to Primary Sclerosing Cholangitis

**DOI:** 10.1002/hep.24299

**Published:** 2011-06

**Authors:** Johannes R Hov, Vasilis Kosmoliaptsis, James A Traherne, Marita Olsson, Kirsten M Boberg, Annika Bergquist, Erik Schrumpf, J Andrew Bradley, Craig J Taylor, Benedicte A Lie, John Trowsdale, Tom H Karlsen

**Affiliations:** 1 Norwegian PSC Research Center, Clinic for Specialized Medicine and Surgery, Oslo University Hospital RikshospitaletOslo, Norway; 2 Research Institute for Internal Medicine, Oslo University Hospital RikshospitaletOslo, Norway; 3 Institute of Immunology, Oslo University HospitalOslo, Norway; 4 Faculty of Medicine, University of OsloOslo, Norway; 5 Tissue Typing Laboratory, Cambridge University Hospitals NHS Foundation Trust, Addenbrooke's HospitalCambridge, United Kingdom; 6 Department of Surgery, University of Cambridge, Addenbrooke's HospitalCambridge, United Kingdom; 7 Division of Immunology, Department of Pathology, University of Cambridge and Cambridge Institute for Medical Research, University of CambridgeCambridge, United Kingdom; 8 Mathematical Sciences, Chalmers University of TechnologyGothenburg, Sweden; 9 Department of Gastroenterology and Hepatology, Karolinska University HospitalHuddinge, Stockholm, Sweden

## Abstract

The strongest genetic risk factors for primary sclerosing cholangitis (PSC) are found in the human leukocyte antigen (HLA) complex at chromosome 6p21. Genes in the HLA class II region encode molecules that present antigen to T lymphocytes. Polymorphisms in these genes are associated with most autoimmune diseases, most likely because they contribute to the specificity of immune responses. The aim of this study was to analyze the structure and electrostatic properties of the peptide-binding groove of HLA-DR in relation to PSC. Thus, four-digit resolution *HLA-DRB1* genotyping was performed in 356 PSC patients and 366 healthy controls. Sequence information was used to assign which amino acids were encoded at all polymorphic positions. In stepwise logistic regressions, variations at residues 37 and 86 were independently associated with PSC (*P* = 1.2 × 10^−32^ and *P* = 1.8 × 10^−22^ in single-residue models, respectively). Three-dimensional modeling was performed to explore the effect of these key residues on the HLA-DR molecule. This analysis indicated that residue 37 was a major determinant of the electrostatic properties of pocket P9 of the peptide-binding groove. Asparagine at residue 37, which was associated with PSC, induced a positive charge in pocket P9. Tyrosine, which protected against PSC, induced a negative charge in this pocket. Consistent with the statistical observations, variation at residue 86 also indirectly influenced the electrostatic properties of this pocket. DRB1*13:01, which was PSC-associated, had a positive P9 pocket and DRB1*13:02, protective against PSC, had a negative P9 pocket. *Conclusion*: The results suggest that in patients with PSC, residues 37 and 86 of the HLA-DRβ chain critically influence the electrostatic properties of pocket P9 and thereby the range of peptides presented. (Hepatology 2011;53:1967-1976)

Genetic predisposition influences the development of primary sclerosing cholangitis (PSC).^1^ The co-occurrence of inflammatory bowel disease and classical autoimmune diseases in patients with PSC suggests that loss of immune tolerance contributes to the pathogenesis. The strongest genetic risk factors in PSC are found in the human leukocyte antigen (HLA) complex at chromosome 6p21.^2^ Many of the genes in this region are immune-related, and variants tend to be inherited together on extended haplotypes, i.e., they are in strong linkage disequilibrium (LD). Deciphering the contribution of the various genes in the region is a major challenge in disease genetics.

As for most HLA-associated diseases, a multitude of HLA class I and class II gene associations have been reported in PSC, most consistently for alleles that are components of the extended ancestral haplotypes AH8.1 (i.e., HLA-B*08-DRB1*03 [serological DR3]) and AH7.1 (i.e., HLA-B*07-DRB1*15 [serological DR2]), along with various less conserved HLA class II haplotypes, namely, DRB1*13:01, DRB1*04, and DRB1*07.^3-6^ In genome-wide association studies,^2^,^7^ strong associations near *HLA-C, HLA-B*, and *MICA* suggest a role for these loci in modifying PSC risk. The mechanism could involve an effect of alleles carried by the AH8.1 and AH7.1 haplotypes on the activation level of natural killer cells and T cells.^8-11^ However, associations detected for HLA class II haplotypes appear to have a significant influence on PSC, in addition to the effect of HLA class I.^7^

The class II genes encode heterodimers consisting of an α and a β chain (e.g., the HLA-DR molecule is encoded by *HLA-DRA* and *HLA-DRB1*) which present peptides to CD4-positive T cells. The sequences encoded by the second exon of class II genes determine the properties of the peptide-binding groove. In several autoimmune diseases HLA class II associations have been attributed to particular amino acids in the molecule that critically determine the binding of disease-specific antigen(s). One example is the protective effect in type 1 diabetes of HLA-DQβ1 chains with aspartic acid in residue 57,^12^ which induces distinct characteristics of the peptide-binding groove of the HLA-DQ molecule.^13^ Determination of the structural and electrostatic properties of the molecules associated with disease may help in identifying the disease mechanism. In primary biliary cirrhosis and autoimmune hepatitis, specific residues have been suggested to explain associations with *HLA-DRB1* alleles.^14^,^15^ In PSC, an association with leucine in residue 38 of the HLA-DRβ chain was proposed by Farrant et al.,^16^ whereas a later study considered residues 55 and 87 of the HLA-DQβ chain as more likely candidates.^3^ A consistent peptide-binding motif for the class II molecules associated with PSC has not been defined, and no attempts have been made to model how specific amino acids affect the structure and the electrostatic properties of the peptide-binding groove.

The portal inflammation in PSC livers is dominated by T cells, which seem to exhibit a restricted T-cell receptor repertoire.^17^ It would be of importance to identify characteristics of the HLA molecules that determine the specificity of these T-cell responses. Strong LD in the HLA class II region makes it difficult to determine at the genetic level which loci are most relevant. However, several minor observations suggest that *HLA-DRB1* could be the determinant of PSC risk; (1) The *HLA-DQA1* and *DQB1* alleles encoded on the AH8.1 haplotype are associated with PSC only on this haplotype and not when encoded on different haplotypes.^4^ (2) The protective DRB1*04 haplotypes may carry different *DQB1* alleles.^4^ (3) A recent study in African-Americans confirms the association with DR13,^18^ which in Northern Europe forms the DRB1*13:01-DQB1*06:03 haplotype,^16^ whereas in African-Americans both DRB1*13:01-DQB1*06:03 and DRB1*13:01-DQB1*05:02 are common haplotypes.^19^ The *HLA-DRB1* association is also more consistent than the association with the closely related (paralogous) *HLA-DRB3* gene; e.g., PSC-associated HLA-DRB1*13:01 haplotypes may carry either the HLA-DRB3*01:01 or DRB3*02:02 alleles.^4^ Given this background we aimed to explore how *HLA-DRB1* variation affects the molecular characteristics of HLA-DR and susceptibility to PSC.

## Materials and Methods

### Subjects

Scandinavian PSC patients (n = 356, Table 1) were recruited from Oslo University Hospital, Rikshospitalet, Oslo, Norway, and Karolinska University, Hospital Huddinge, Stockholm, Sweden. Diagnosis of PSC was based on accepted criteria with typical cholangiographic appearance. Ethnically and gender-matched healthy controls (n = 366) were randomly selected from the Norwegian Bone Marrow Registry. All participants gave informed consent. The study was approved by the Regional Committee for Research Ethics in South-Eastern Norway and the Ethics Committee of Karolinska Institutet.

**Table 1:** Characteristics of Included Individuals

	PSC	Healthy Controls
N	356*	366
Male, n (%)	254 (71)	256 (70)
Age at diagnostic cholangiography, years, median (range)	36 (12-75)	—
Concomitant inflammatory bowel disease, n (%)	290† (82)	—
Ulcerative colitis/Crohn's disease/indeterminate, %	81/12/7	—
Cholangiocarcinoma, n (%)	50 (14)	—
Endpoint (tx or death), n (%)	210 (59)	—
Follow-up, years, median (range)	10 (0-34)	—

* Norway n = 230, Sweden n = 126.

† Missing information about the intestine in three patients.

### *HLA-DRB1* Data

Four-digit *HLA-DRB1* genotypes were available from a previous study.^20^ Peptide sequences of all *HLA-DRB1* alleles in IMGT/HLA database release 2.23 (October 2008) were aligned, and each individual was assigned two amino acids (one encoded by each chromosome) for each polymorphic residue.

### Statistical Methods

Stepwise logistic regressions were performed in the statistical package R v2.10.0 (http://www.r-project.org/) assuming an “allele dosage” model, entering the count of all amino acids at a given residue as covariates. A model with all observed combinations of amino acids (“genotypes”) at a given residue entered as covariates was applied to control the validity of the model. Some combinations of amino acids were rare and after testing several criteria, combinations with a frequency of n < 2 in cases or controls at a given residue were grouped in order to avoid empty cells. In both models the reference was randomly chosen, thus no assumptions were made on which amino acid or pair of amino acids constituted high or low risk. Comparisons of allele and carrier frequencies were performed in Microsoft Excel (Redmond, WA) and PASW v. 18 (SPSS, Chicago, IL). *P* < 0.05 was considered statistically significant. *P*-values of novel *HLA-DRB1* allele associations were Bonferroni corrected according to the number of alleles present in the dataset (n = 32).

### 3D Protein Structure Modeling of HLA-DR Molecules

The atomic coordinates of the most common HLA-DR molecules were determined using comparative protein structure modeling by satisfaction of spatial restraints as implemented in the MODELLER computer algorithm.^21^ HLA-DR proteins of known structure suitable as modeling templates were identified in the Protein Data Bank (PDB; http://www.rcsb.org/pdb/) and evaluated for structural quality. Accordingly, seven structures were selected as templates (PDB entries: 1KLU, 2G9H, 1D5Z, 1D5M, 2Q6W, 1PYW, and 2IPK). The amino acid sequences of the target HLA-DR molecules were obtained from the IMGT/HLA database. Multiple sequence alignments were performed with CLUSTAL_X v.1.83^22^ and manually corrected when indicated. The alignment files were then used as input to the MODELLER program. In brief, MODELLER generates the 3D atomic coordinates of the target sequences by satisfying spatial restraints, obtained from the templates, and by CHARMM^23^ energy terms enforcing proper stereochemistry. Optimization is then carried out by employing methods of conjugate gradients and molecular dynamics with simulated annealing.^24^ All calculations were performed in the absence of antigenic peptides to enable direct comparison of the structural and physiochemical characteristics of the peptide-binding groove among different molecules. The stereochemical quality of the modeled structures was verified using the PROCHECK^25^ and WHAT_CHECK^26^ algorithms and by assessment of Ramachandran plots. In addition, the structures were examined for protein folding quality using empirical energy potentials as implemented in the ProSA algorithm.^27^ Modeled coordinate sets are available upon request.

### Electrostatic Potential Calculations

The electrostatic potential around the 3D structures was computed by numerically solving the Poisson Boltzmann equation using the finite difference method implemented in the DelPhi program within Discovery Studio 2.1 (Accelrys, San Diego, CA). Essential hydrogens were added to the structures. To determine the protonation state of titratable amino acid side chains the titration curves and residue pKa were calculated for each molecule (dielectric constant of 10 for the protein interior and 80 for the solvent) and titratable residues were protonated at a pH of 7.4. The protonated protein molecule was subsequently used to compute the electrostatic potential. The low dielectric protein interior (dielectric constant of 2) was embedded in a high dielectric continuum environment (water exterior, dielectric constant of 80). A solution with charged ions was simulated with an assigned ionic strength of 0.145, typical of the conditions at a pH of 7.4. The dielectric boundary between the protein and the solvent was defined by calculating the solvent-accessible surface generated by a rolling probe sphere of 1.4 Å radius. Atomic radii and partial atomic charges were taken from the CHARMM parameter set.^23^ An ion exclusion layer (Stern layer) for the solvent ions was defined around the solvent-accessible surface using an ionic radius of 2 Å. The layer has an ionic strength of 0.0 and determines the maximum distance that an ion can approach the solvent-accessible surface. The system was mapped into a 3D cubical grid and the electrostatic potential at each grid point was calculated iteratively starting from the Debye-Hückel boundary conditions. The accuracy of the calculations was improved by using a method of grid focusing; in the first run the coarse grid was allowed to be filled by 50% by solute and the calculated grid point potentials were used in the second run where the fine grid was filled by solute by 90%. The grid dimensions were set at 251 grid points per axis (spacing 0.3 Å / grid point). The solvent accessible surface was colored according to its calculated electrostatic potential and visualized using the Discovery Studio interface.

## Results

### Statistical Modeling: Identification of Position 37 and 86 in the DRβ1 Chain as PSC-Associated Residues

The amino acid sequence encoded by exon two of *HLA-DRB1* was determined from the genotypes of each individual. Thirty residues were polymorphic, i.e., two or more different amino acids were observed at these positions. In the first step, a logistic regression was performed for each polymorphic residue. The counts (0, 1, 2) of the observed amino acids were included as covariates and the overall effect of the residue was tested with a likelihood ratio test. The strongest PSC associations were detected for residue 37 (*P* = 1.2 × 10^−32^, Table 2). In a second step, two-residue models were fitted containing the amino acid covariates of both the investigated residue and residue 37 and compared with the single-residue model of residue 37. The only residue that remained strongly associated with PSC in these two-residue models was 86 (Table 2). When performing a similar two-residue test for additional effects on top of 86, several residues (i.e., also residue 37) were found to contribute significantly (Table 2). No other residues showed significant disease association when included in three-residue models with residues 37 and 86 (Table 2).

**Table 2:** Association Analyses Between Amino Acid Variation in the HLA-DRβ1 Chain and PSC*

		Single-Residue LR		Two-Residue LR†				Three-Residue LR‡
				
		Basic Model: -		Basic Model: Residue 37		Basic Model: Residue 86		Basic Model: Residues 37+86
					
Residue	Observed Amino Acids	*P*-value	Rank	*P*-value	Rank	*P*-value	Rank	*P*-value
9	Glu, Lys,Trp	0.051	27	0.59	22	0.0086	20	0.29
10	Glu,Gln,Tyr	2.4 × 10^−11^	14	0.54	21	0.0017	17	0.48
11	Asp,Gly,Leu,Pro,Ser,Val	1.2 × 10^−18^	8	0.23	15	1.7 × 10^−8^	3	0.43
12	Lys,Thr	2.7 × 10^−12^	12	0.64	24	0.00046	14	0.74
13	Phe,Gly,His,Arg,Ser,Tyr	9.6 × 10^−25^	2	0.22	14	2.1 × 10^−11^	2	0.25
14	Glu,Lys	0.0014	20	0.36	17	0.74	28	0.83
16	His,Tyr	3.2 × 10^−5^	17	0.88	29	0.00053	15	0.78
25	Gln,Arg	0.0014	21	0.36	18	0.74	29	0.83
26	Phe,Leu,Tyr	2.6 × 10^−19^	6	0.024	2	3.9 × 10^−8^	4	0.23
28	Asp,Glu,His	2.8 × 10^−5^	16	0.13	8	0.0053	19	0.10
30	Cys,Gly,His Leu,Arg,Tyr	0.00012	18	0.12	7	2.0 × 10^−5^	11	0.20
31	Phe,Ile,Val	0.31	29	0.078	5	0.0046	18	0.12
32	His,Tyr	1.2 × 10^−21^	4	0.38	19	6.6 × 10^−8^	6	0.64
33	His,Asn	1.4 × 10^−15^	11	0.40	20	5.0 × 10^−8^	5	0.31
37	Phe,Leu,Asn,Ser,Tyr	**1.2 × 10^−32^**	**1**	—		**3.1 × 10^−16^**	**1**	—
38	Ala,Leu,Val	0.029	26	0.17	11	0.0014	16	0.14
40	Phe,Tyr	0.77	30	0.17	12	0.52	27	0.14
47	Phe,Tyr	2.4 × 10^−21^	5	0.11	6	0.00024	13	0.29
57	Ala,Asp,Ser,Val	2.6 × 10^−5^	15	0.78	26	0.16	22	0.44
58	Ala,Glu	0.00055	19	0.85	28	0.035	21	0.84
60	His,Ser,Tyr	0.0046	24	0.59	23	0.28	24	0.29
67	Phe,Ile,Leu	0.0018	22	0.80	27	0.45	25	0.22
70	Asp,Gln,Arg	0.063	28	0.16	10	0.49	26	0.32
71	Ala,Glu,Lys,Arg	1.4 × 10^−15^	10	0.043	3	5.4 × 10^−6^	8	0.32
73	Ala,Gly	5.5 × 10^−12^	13	0.14	9	1.7 × 10^−5^	10	0.34
74	Ala,Glu,Leu,Gln,Arg	1.7 × 10^−18^	9	0.25	16	9.4 × 10^−6^	9	0.59
77	Asn,Thr	5.8 × 10^−19^	7	0.060	4	4.8 × 10^−7^	7	0.34
78	Val,Tyr	0.023	25	0.77	25	0.25	23	0.20
85	Ala,Val	0.0037	23	0.21	13	7.2 × 10^−5^	12	0.13
86	Gly,Val	1.8 × 10^−22^	3	**2.0 × 10^−5^**	**1**	—		—

* Stepwise logistic regressions were performed assuming an “allele dosage” effect.

† *P*-values of likelihood ratio tests of whether residue n improves the logistic regression model when added to a model with one other residue (37 or 86).

‡ *P*-values of likelihood ratio tests of whether residue n improves the model when added to a model with both residues 37 and residue 86.

LR: likelihood ratio; Rank: residue rank according to *P*-value, lowest *P*-value is highlighted in bold.

In the logistic models used above the effect of a single amino acid was assumed to be additive on the log-scale: The log-odds ratio of having PSC given two copies of the amino acid is two times the log-odds ratio when having one copy. The advantage with this model is that it keeps the number of covariates to a minimum, leading to more powerful tests as long as the model assumptions are approximately true. In order to confirm the results obtained with this model, we also performed regressions where we allowed each observed combination (“genotype”) of amino acids to have a potential effect. In these “genotype” model analyses, residue 37 remained the most significantly PSC-associated residue (*P* = 6.9 × 10^−32^, Table 3), with an independent contribution from residue 86 still observed (*P* = 1.2 × 10^−5^). Several other residues contributed on top of residues 37 or 86 in two-residue models, as well as in three-residue models with both residues 37 and 86 included (Table 3). When inspecting the distribution of amino acid combinations (“genotypes”) in the dataset, it became apparent that the extra associated residues 26, 70, 71, 73, 74, and 77 (Table 3) reflected a large number of patients homozygous for HLA-DRB1*03:01 (n = 62 patients versus n = 3 healthy controls), meaning that it was not possible to determine the part of HLA-DRB1*03:01 that confers this additional risk.

**3:** Summary of Association Analyses Between Amino Acid Variation and PSC Assuming a “Genotype” Model*

	Single-Residue LR	Two-Residue LR†,				Three-Residue LR‡
			
	Basic Model: -	Basic Model: Residue 26	Basic Model: Residue 37	Basic Model: Residues 77	Basic Model: Residues 86	Basic Model: Residues 37 + 86
						
Residue	*P*-value	*P*-value	*P*-value	*P*-value	*P*-value	*P*-value
26	6.2×10^−20^	—	4.2×10^−7^	0.19	1.3×10^−8^	4.1×10^−5^
37	6.9×10^−32^	3.3×10^−19^	—	1.5×10^−19^	4.8×10^−17^	—
70	0.013	0.23	0.023	0.083	0.055	0.036
71	1.6×10^−17^	3.8×10^−14^	0.00034	3.4×10^−13^	7.1×10^−8^	0.014
73	1.1×10^−12^	0.15	0.00075	0.17	8.1×10^−6^	0.0038
74	1.6×10^−18^	0.14	6.0×10^−5^	0.096	1.3×10^−6^	0.00093
77	7.3×10^−21^	0.80	8.5×10^−8^	—	7.8×10^−9^	3.4×10^−6^
86	2.0×10^−21^	1.3×10^−9^	1.2×10^−5^	2.2×10^−9^	—	—

* Stepwise logistic regressions were performed assuming a “genotype” effect, entering all different pairs (combinations) of amino acids at a given residue. Only residues contributing significantly to the model when added to residue 37 and 86 in the three-residue regressions are shown.

† *P*-values of likelihood ratio tests of whether residue n improves the logistic regression model when added to a model with one other residue (26, 37, 77, or 86).

‡ *P*-values of likelihood ratio tests of whether residue n improves the model when added to a model with both residues 37 and residue 86.

LR: likelihood ratio.

In conclusion, residues 37 and 86 were consistent determinants of PSC susceptibility irrespective of statistical model, whereas it was difficult to exclude additional risk associated with other parts of the β chain encoded by HLA-DRB1*03:01.

### Residue 37 Influences the Electrostatic Properties of Pocket P9 of HLA-DR

The amino acid frequencies at residues 37 (Table 4) showed that the highest and lowest risks of PSC were observed for carriers of asparagine (Asn37) (odds ratio [OR] = 5.7, 95% confidence interval [CI] 4.0-8.0) and tyrosine (Tyr37) (OR = 0.25, 95% CI 0.18-0.34), respectively.

**Table 4:** Frequencies of Different Amino Acids at HLA-DRβ1 Residues 37 and 86

		Allele Frequency, n (%)		Carrier Frequency, n (%)	
			
Residue	Amino Acid	PSC	Healthy Controls	PSC	Healthy Controls
37	Asparagine (Asn)	398 (56)	199 (27)	292 (82)	163 (45)
	Leucine (Leu)	4 (1)	16 (2)	4 (1)	15 (4)
	Phenylalanine (Phe)	35 (5)	67 (9)	34 (10)	62 (17)
	Serine (Ser)	192 (27)	208 (28)	162 (46)	175 (48)
	Tyrosine (Tyr)	83 (12)	242 (33)	80 (22)	197 (54)
86	Glycine (Gly)	185 (26)	368 (50)	164 (46)	284 (78)
	Valine (Val)	527 (74)	364 (50)	335 (94)	282 (77)

**Fig. 1:**
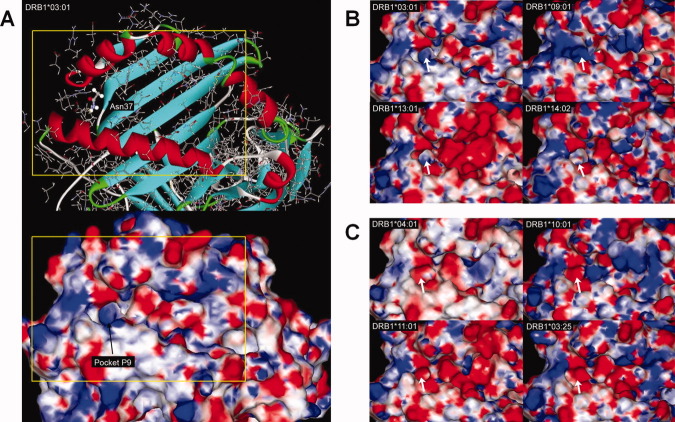
Structure and molecular surface electrostatic potential of pocket P9. (A) The structure and electrostatic potential of HLA-DRB1*03:01. The area within the frame is depicted in expanded form in (B,C). All structures were superimposed on HLA-DRB1*03:01 and therefore show the same view. HLA-DR carrying the risk residue Asn37 in the β chain had P9 pockets (arrows) with positive charge (B), whereas molecules expressing Tyr37 had P9 pockets (arrows) with consistently negative charge (C). Potentials less than −5 kT/e are colored red, those greater than 5 kT/e blue, and neutral potentials (0 kT/e) are colored white. Linear interpolation was used to produce the color for surface potentials between these values.

The specificity of the peptide-binding groove on an HLA class II molecule is governed by the properties of pockets in the groove that accommodate the amino acid side chains of the bound peptide, typically pockets for peptide residues 1 (pocket P1), 4, 6, and 9. Residue 37 of the HLA-DRβ1 chain is integral to pocket P9.^28^ Figure 1 shows the structural and electrostatic characteristics of pocket P9 on representative HLA-DR molecules. Significantly, HLA-DR carrying the risk residue Asn37 in the β chain (e.g., HLA-DRB1*03:01, *09:01, *13:01, *14:02; Fig. 1B) formed P9 pockets with similar structural architecture and consistently positive surface electrostatic potential (the only exception was HLA-DRB1*13:02, further discussed below). In contrast, HLA-DR molecules expressing the protective Tyr37 residue in the β chain (e.g., HLA-DRB1*04:01, *10:01, *11:01, *03:25; Fig. 1C) formed P9 pockets with consistently negative electrostatic potential. The distinct P9 pocket electrostatic patterns were conserved both among molecules that differed at several amino acid sequence positions and between structures where residue 37 constitutes the only disparity (e.g., HLA-DRB1*03:01 and -DRB1*03:25). Interestingly, a database search for peptides eluted from HLA-DR molecules showed that the presence of Asn37 restricted the amino acid preferences at position 9 (e.g., only tyrosine, leucine, and phenylalanine are defined as P9 anchors in HLA-DRB1*0301), whereas most amino acids may be P9 anchors in HLA-DRB1*0401 which carries Tyr37 (http://www.syfpeithi.de).^29^

### Residue 86 Defines Opposite Effects of HLA-DRB1*13:01 and *13:02 on PSC Risk

**Fig. 2:**
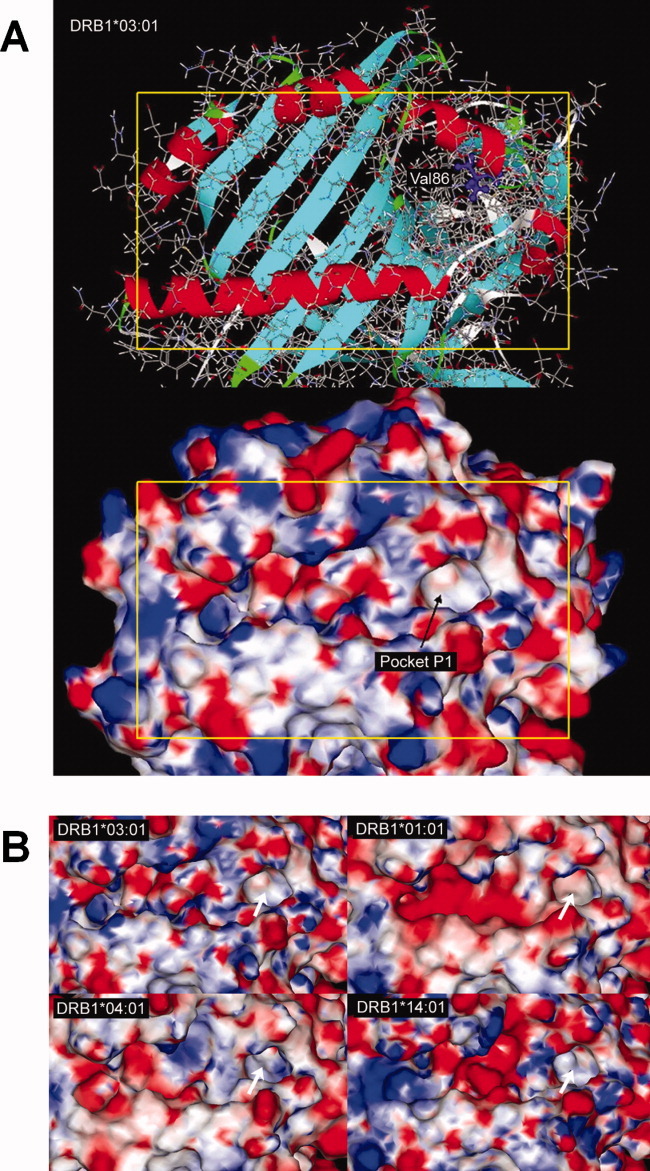
Structure and molecular surface electrostatic potential of pocket P1. (A) The structure and electrostatic potential of HLA-DRB1*03:01. The area within the frame is depicted in expanded form in (B). All structures were superimposed on HLA-DRB1*03:01 and therefore show the same view. Structural modeling and calculation of the electrostatic potential at the P1 pocket (arrows) of representative HLA-DR molecules showed that the Gly/Val dimorphism at position 86 had a minimal physiochemical effect (B). The majority of HLA-DR molecules examined had P1 pockets with an overall neutral charge. HLA-DRB1*03:01 and -DRB1*14:01 express Val86 whereas -DRB1*01:01 and -DRB1*04:01 express Gly86. Potentials less than −5 kT/e are colored red, those greater than 5 kT/e blue, and neutral potentials (0 kT/e) are colored white. Linear interpolation was used to produce the color for surface potentials between these values.

At the dimorphic residue 86, the highest risk was observed for carriers of valine (Val86) (OR = 4.8, 95% CI 2.9-7.9), whereas glycine (Gly86) appeared protective (OR = 0.25, 95% CI 0.18-0.34). Residue 86 of the HLA-DRβ1 chain is integral to pocket P1.^28^ In contrast to pocket P9, modeling of the P1 pocket of several HLA-DR molecules showed that the glycine/valine dimorphism at residue 86 had a minimal physiochemical effect. The majority of HLA-DR molecules examined had P1 pockets with an overall neutral charge (Fig. 2). Even though a steric effect (i.e., an effect on the volume of the pocket) imposed by the side chain of Val86 cannot be excluded, the results of the present analysis argue against a significant role of residue 86 on the choice of peptide residue accommodated by pocket P1.

**Fig. 3:**
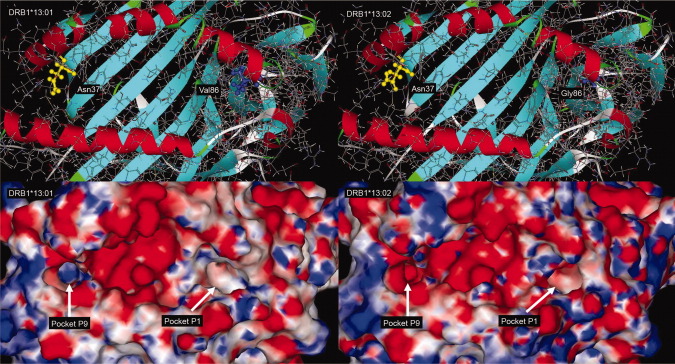
Electrostatic modification of pocket P9 by the Gly86-to-Val86 substitution. The structure and electrostatic potential of the peptide-binding groove is shown for (structures were superimposed) HLA-DRB1*13:01 (left figures) and -DRB1*13:02 (right figures); these molecules have a single amino acid sequence difference at position 86 (Val86 and Gly86, respectively). HLA-DRB1*13:02 has an electronegative pocket P9 despite the presence of Asn at position 37, suggesting a long-range effect of the Val86-to-Gly86 substitution. The molecular surface is colored according to the calculated electrostatic potential, as for Figs. 1 and 2.

Further analysis, however, led to an interesting observation. As mentioned above, HLA-DR molecules expressing the risk residue Asn37 in their β chain possess electropositive P9 pockets, with the exception of HLA-DRB1*13:02 where an electronegative P9 pocket was observed (Fig. 3). Notably, when looking at the allele frequencies, HLA-DRB1*13:02, as opposed to other Asn37 encoding alleles (like the established PSC risk allele HLA-DRB1*13:01), was more frequent in healthy controls than in PSC patients (*P*_corrected_ = 0.040, Table 5), suggesting that HLA-DRB1*13:02 may protect against PSC. This statistical observation is therefore in agreement with the protective effect associated with HLA-DR molecules expressing electronegative P9 pockets, as shown above for Tyr37 encoding alleles. Intriguingly, HLA-DRB1*13:02 and DRB1*13:01 have otherwise overall similar structural architecture and electrostatic properties (Fig. 3) with the main disparity observed at pocket P9. Because the only amino acid sequence difference between these alleles is at position 86 it may be suggested that the Gly86Val substitution may influence the choice of presented peptides through long-range electrostatic modification of pocket P9.

**Table 5:** Frequency of *HLA-DRB1*Alleles Encoding Asn37

	PSC (2n=712)		Healthy Controls (2n=732)				
					
Allele	n	(%)	n	(%)	OR (95%CI)*	*P*-value†	Residue 86
03:01	254	(36)	106	(14)	3.3 (2.5-4.2)	1.3 × 10^−20^	Val
09:01	12	(2)	6	(1)	2.0 (0.8-5.0)	0.14	Gly
13:01	117	(17)	47	(6)	2.8 (2.0-4.1)	2.0 × 10^−9^	Val
13:02	15	(2)	39	(5)	0.4 (0.2-0.7)	0.0013‡	Gly
14:02	0	(0)	1	(0)	0.3 (0.0-3.8)	1.0	Gly

* Calculated with Woolf's formula with Haldane's correction.

† Not corrected. Calculated with chi-square tests or Fisher's exact test where appropriate.

‡ *P*_corrected_ = 0.040.

OR = odds ratio. CI = confidence interval.

## Discussion

By exploring variation in the amino acid sequence of the HLA-DRβ1 chain in PSC, we show that residues 37 and 86 distinguish disease susceptibility alleles and protective alleles. Investigations into the HLA-DR molecular structure revealed that the electrostatic properties of pocket P9 are determined by residue 37 and, indirectly by residue 86, suggesting that the P9 pocket is crucial for PSC risk.

In the HLA-DR molecule, residue 37 of the β chain appeared to be a key determinant of the electrostatic properties of pocket P9, which may be related to disease risk. The situation is reminiscent of type 1 diabetes, where amino acids at residue 57 of the HLA-DQ β chain associated with disease risk contribute to a larger volume of pocket P9 and a positive charge, allowing, e.g., glutamate residues from insulin peptides at position 9.^30^ In HLA-DR, Asn37 would restrict the range of amino acids at anchor position 9 of the peptide, and thereby which peptides may be presented. This is supported by data from peptide elusion experiments, where HLA-DR molecules with Asn37 and Tyr37 exhibit different ranges of amino acids at P9.^29^ Direct experimental observations focusing on pocket P9 variation and T-cell responses are scarce, but it has been shown that modification of only residue 37 (on DR4 molecules) is sufficient to alter recognition by the T-cell receptor, e.g., by neutralizing the T-cell-activating potential of the peptide-DR-complex.^31^,^32^ It should therefore be considered highly likely that characteristics of pocket P9 of the HLA-DR molecule facilitate particular immune responses.

Pocket P1 of HLA-DR was found to have an overall neutral electrostatic potential in the present study irrespective of whether glycine or valine was present at position 86. This fits with the observation that this pocket has a preference for hydrophobic amino acid side chains, and that the range of amino acids in position 1 of presented peptides is largely overlapping.^33^,^34^ However, pocket P1 with Gly86 in the β chain (e.g., as encoded by HLA-DRB1*13:02) has a tendency to accept larger (aromatic) side chains than when Val86 is present (e.g., encoded by DRB1*13:01); this has been attributed to the lack of a side chain on glycine allowing for a larger pocket volume.^33^,^34^ A more remarkable difference between HLA-DRB1*13:01 and DRB1*13:02 encoded HLA-DR molecules was that the amino acid substitution at residue 86 affected the electrostatic properties of pocket P9, in another part of the molecule. HLA-DRB1*13:02 was the only allele which contributed to a HLA-DR molecule with a negative pocket P9 with asparagine at position 37 of the β chain. Intriguingly, this allele exhibited a significantly reduced frequency in PSC patients. Taken together, our findings suggest that the association of residues 37 and 86 with PSC primarily reflects the properties of pocket P9.

Although HLA-DRB1*13:01 is a well-established PSC risk allele,^4^ this study is the first identifying HLA-DRB1*13:02 as a protective allele. This observation was significant even when correcting for multiple comparisons. Interestingly, similar contrasting effects have been observed in autoimmune hepatitis in Latin America, where risk is associated with HLA-DRB1*13:01 and protection with DRB1*13:02.^15^ HLA-DRB1*13:01 has also been associated with a protracted course of hepatitis A virus infection, which has been postulated to be a trigger of autoimmune hepatitis.^35^ To what extent these parallel observations are relevant for the specificity of the immune response in PSC can currently only be speculated.

Given the complexity of the HLA associations in diseases such as PSC, it is not unlikely that other alleles besides the most strongly associated ones modify the disease risk. Two previous studies of HLA-DR in PSC evaluated selected residues encoded by haplotypes associated with disease,^3^,^16^ and suggested that the presence of leucine at position 38 (Leu38) of the β chain may confer risk. Leu38 is rarely present in DRβ1 (most often encoded by DRB1*12 alleles). An explanation for the conflicting results is that the previous studies included alleles at both the *HLA-DRB1* locus as well as those at other, paralogous, HLA-DRB loci. Several HLA haplotypes carry a second HLA-DRB gene besides *HLA-DRB1*, e.g., HLA-DRB1*03:01 and *13:01 haplotypes typically also carry an allele encoded by *HLA-DRB3*; DRB1*04 and *07:01 carry an allele encoded by *HLA-DRB4*, and the DRB1*15:01 haplotype carries an allele encoded by *HLA-DRB5*. These β chains couple with DRα and also have a role in antigen presentation.^36^ They are generally observed at several-fold lower expression levels than DRβ1.^37^,^38^ However, in diseases where the second DRB gene has been shown to be of actual relevance, the association seems to be specific to the gene in question and not due to shared sequence motifs with DRB1.^39^–^41^ These facts, along with the more consistent PSC associations with *HLA-DRB1* rather than *HLA-DRB3*,^4^ make it likely that the present focus on *DRB1* is valid, even though an effect of other *DRB* loci cannot be formally ruled out at this stage.

Given the LD in the HLA complex, we cannot exclude the possibility that causal variants at other loci may be associated with the distribution of amino acids observed at given positions in HLA-DRβ1. The strong LD is particularly important in relation to the neighboring *HLA-DQ* genes and *HLA-DRB* paralogs, but it is also difficult to formally exclude an association with the nearby *BTNL2* gene, which has been associated with inflammatory bowel disease,^42^ or even genetic variants further away. When applying a “genotype” model, in addition to residue 37 and 86 we could not exclude a residual association that could be attributed to being homozygous for HLA-DRB1*03:01. This may be speculated to relate to effects of a recessive variant outside *HLA-DRB1*,^4^ potentially related to the AH8.1 haplotype which is associated with multiple autoimmune diseases and probably contains several genetic variants in strong LD contributing to disease.^43^

In conclusion, this study shows that variation in PSC associated residues encoded by *HLA-DRB1* impose distinct structural and physiochemical characteristics on the HLA-DR peptide-binding groove, suggesting that PSC risk molecules likely present a restricted peptide repertoire. The findings are highly relevant for and important to evaluate in future experimental studies of antigen presentation in PSC. The amino acid sequence and structural observations did not apply uniformly to all PSC patients, suggesting multiple pathogenetic mechanisms, as might be expected for a disease with the clinical heterogeneity observed in PSC.

## Abbreviations

AH: ancestral haplotype
HLA: human leukocyte antigen
LD: linkage disequilibrium
PSC: primary sclerosing cholangitis
